# Protective Effect of Fish Oil Supplementation on DNA Damage Induced by Cigarette Smoking

**DOI:** 10.3329/jhpn.v31i3.16826

**Published:** 2013-09

**Authors:** Amir Ghorbanihaghjo, Javid Safa, Samira Alizadeh, Hassan Argani, Nadereh Rashtchizadeh, Mohammad Vahid Taghinia, Mehran Mesgari Abbasi

**Affiliations:** ^1^Biotechnology Research Center, Tabriz University of Medical Sciences, Tabriz, Iran; ^2^Drug Applied Research Center, Tabriz University of Medical Sciences, Tabriz, Iran

**Keywords:** 8-hydroxy-2’-deoxyguanosine, Antioxidants, Cigarette smoking, Fish oil

## Abstract

The study examined the influence of fish oil (FO) supplementation on serum 8-hydroxy-2’-deoxyguanosine (8-OHdG) levels as indicated by DNA damage markers and total antioxidant capacity (TAC) among male cigarette smokers. This double-blind, placebo-controlled randomized study was conducted among healthy cigarette smokers (n=40) who were part of a larger prospective cohort study. Twenty smokers were randomly selected to receive FO for 3 months (1 g/day), and another 20 smokers received a placebo for 3 months; 8-OHdG and TAC levels were measured in blood samples before and after the intervention. Serum 8-OHdG significantly decreased (p=0.001) and TAC increased (p<0.001) after 3 months of treatment with FO. Between baseline and endline, the difference in 8-OHdG significantly correlated with the difference in TAC among smokers who received FO (r=-0.540, p=0.014). The study provides evidence that FO supplementation can modify decreased antioxidants and increased oxidative DNA damage in cigarette smokers.

## INTRODUCTION

8-hydroxy-2’-deoxyguanosine (8-OHdG) is a product of oxidative DNA damage induced by the action of hydroxyl radicals on the DNA base deoxyguanosine (dG) (1,2) and DNA single-strand breakage (3,4). Damage to DNA may lead to the initiation of carcinogenesis. Evidence suggests that 8-OHdG is reflective of the potentially-precancerous disease processes. However, the precise predictive value of 8-OHdG for the development of cancer remains obscure (5). A decrease in 8-OHdG would show a decrease in oxidative damage to DNA (6).

Several studies have examined the effects of dietary intake of antioxidant nutrients, such as vitamin E, vitamin C, and ß-carotene on biomarkers of oxidative stress (7). As dietary supplementation with n-3 fatty acids may diminish the release of free radicals from stimulated human monocytes and polymorphonuclear cells, changes in the cellular free radical production due to omega-3 fatty acids may, in turn, influence cell-mediated oxidative modifications of DNA *in vivo* (8).

Some studies, with contradictory results, have evaluated the effect of omega-3 fatty acids on oxidative DNA damage in animal and human models (9). Manna S *et al*. (10) reported in an *in vivo* study that n-3 polyunsaturated fatty acids (PUFAs) protect against the generation of DNA-strand breaks in rat mammary carcinogenesis. However, some reports have indicated that lipid peroxides that arise from both spontaneous and enzymatic oxidation of polyunsaturated fatty acids are the major source of endogenous DNA damage linked to various age-related pathologies and initiating carcinogenesis (11).

Cigarette smoke contains approximately 1×l0^15^ free radicals per puff (12) and is believed to induce oxidative stress in the body through several mechanisms, including direct damage by free radicals and inflammatory responses. Peroxyl radicals and reactive nitrogen species (13), including NO, ONOO^-^, and ROONO (1), directly damage (13) biological systems by stimulating lipid peroxidation (6,13) and oxidizing and nitrating proteins, lipids, and DNA bases (13). Relatively high amounts of these free radicals and other oxygen-derived species in cigarette smoke can deplete antioxidants, modify proteins and amino acids, promote atherosclerotic disease, and oxidize lipoproteins, particularly low-density lipoprotein (LDL) which is more atherogenic than native LDL (7,14). The hydroquinone/quinine complexes of tar-phase cigarette smoke diffuse across cell membranes and produce semiquinones, superoxide radicals (O_2_^-^), and hydrogen peroxide (H_2_O_2_) (3). Smoking causes inflammatory reactions in the lungs, which may lead to elevated oxidative stress and pulmonary diseases (15).

Epidemiological studies have shown that cigarette smoking is a main contributory factor for biomolecular damage, tissue injuries, and long-term associations with cigarette smoke-related diseases. Nearly 50% of the deaths in the industrialized world result from coronary artery disease. There is increasing evidence that cigarette smoking contributes considerably to this mortality (3,12). The available evidence also demonstrates that cigarette smoke induces the formation of 8-OHdG (1-4). It has been demonstrated that smokers are exposed to high concentrations of oxidants and free radicals from both gas-phase cigarette smoke and particulate matter (14,16). These free radicals are a major cause of oxidative damage to macromolecules, such as lipids, proteins, and DNA, and deplete some plasma antioxidants *in vitro* (17). Additionally, as polyunsaturated fatty acids (PUFAs) are highly susceptible to free radical oxidation, current smoking may reduce the amount of PUFAs in plasma and red blood cells (18). Therefore, smoking cigarettes may lead to an enhanced requirement for PUFAs. Long-chain polyunsaturated omega-3 fatty acids, such as docosahexaenoic acid (DHA) and eicosapentaenoic acid (EPA), that are mainly found in fish oil have been shown to reduce the risk of age-related macular degeneration (19) and chronic obstructive pulmonary disease (20) in cigarette smokers.

It is reported that smokers frequently have lower levels of antioxidant capacity in their blood compared to non-smokers (7,14). Moreover, data extracted from the Continuing Survey of Food Intakes by Individuals (CSFII) suggest that habitual smokers consume the least antioxidant nutrients (5). Since reactions of radicals could be involved in smoking-related diseases and deaths due to smoke inhalation (3), and components of the diet may modify the effect of smoking on oxidative damage to tissues, it is generally assumed that dietary antioxidants may attenuate some of the deleterious effects associated with cigarette smoking (15).

To our knowledge, no studies have determined the effects of dietary fish oil on oxidative stress among cigarette smokers.

The objective of this study is to show whether fish oil supplementation (rich in omega-3 fatty acids) is capable of decreasing serum 8-hydroxy-2’-deoxyguanosine as a DNA damage marker and increasing TAC as a tool to assess redox status in male cigarette smokers.

## MATERIALS AND METHODS

### Participants

Participants in the study were selected from a previous prospective cohort of long-term smokers (n=40) and age-matched controls who had never smoked (n=40) between 2008 and 2009. In this randomized control trial, we included those cigarette smokers who had regularly smoked 10 or more cigarettes per day consecutively for at least 2 years prior to the study. All subjects who had been taking vitamin and micronutrient supplements (e.g. antioxidants) in the previous 2 months and during the experiment period were excluded. Major exclusion criteria for entry into the study included intake of alcohol in the past two years and during the study period, coronary heart disease, diabetes mellitus, thyroid disorders, other metabolic diseases, pulmonary disorders, cancer, or renal disease. During an initial screening visit, information on the smoking habits, personal details, drug consumption and health history of the subjects were collected by verbal communication. The intake of various food-items and frequency of consumption per day over the past year were assessed through interviews and with the help of a structured food frequency questionnaire.

In the second phase, as part of a double-blind, placebo-controlled study, cigarette smokers were randomly divided into two groups: 20 smokers received FO (1 g/day) for 3 months, and 20 smokers received placebo for 3 months. Forty non-smokers also were randomly divided into two equal groups and received FO (1 g/day) and placebo for 3 months. Each capsule (1 g) of FO (provided by Zahravi Co.) was composed of 180 mg of eicosapentaenoic acid (EPA, 20:5 ω-3) and 120 mg of docosahexaenoic acid (DHA, 22:6 ω-3). The placebo capsules were similar to the FO capsules, with the exception of ω-3. Participants were instructed to consume single daily doses of capsules in empty stomach (fasting state). Daily ingestion of FO capsule in a fasting state was being monitored by trained nurses when giving the capsules every morning. Any health-related events during the study period and continuity of cigarette smoking in the smoker group and absence of cigarette smoking in the other group were monitored every day and excluded from the study if it was necessary.

All experiment procedures were conducted according to the Helsinki Declaration and approved by the Ethics Committee of the Tabriz University of Medical Sciences. All participants received verbal and written information regarding potential risks and the end-points of the study. Signed informed consent was obtained from each subject before participation in the study.

### Sample collection and analysis

About 3 mL of venous blood sample was obtained by trained nurses from each subject after overnight fasting both at the start of the study and at the end of treatment with FO or placebo. Aliquots of the sera were separated and stored at −70 °C until laboratory tests were performed.

TACs of the samples were measured by a spectrophotometric assay with Randox total antioxidant status kit. For this method, incubation of 2, 2´ -azino-di (3-ethylbenzthiazoline sulphonate), ABTS, with a peroxidase (metmyoglobin) results in the production of the cation radical ABTS^+^. This species is blue-green in colour and can be detected at 600 nm. Any antioxidants in the added sample inhibit this colour production in proportion to their concentration. Serum 8-OHdG concentration was measured by using a commercially-available ELISA kit (8-OHdG Check–Japan Institute for the Control of Aging), that utilizes monoclonal antibody which is specific for DNA damage with detection range of 0.5 ng/mL-200 ng/mL. The standard curve concentrations used for the ELISA were 200 ng/mL, 80 ng/mL, 20 ng/mL, 8 ng/mL, 2 ng/mL and 0.5 ng/mL.

### Statistical analysis

Statistical analyses were performed using SPSS (version 15) for Windows (SPSS, Inc., Chicago, IL). The Kolmogorov–Smirnov test was used in evaluating the distributions, and the results were expressed as mean±SD. Mann–Whitney U Test and Independent Sample *t*-tests for unpaired data and Wilcoxon and Student's *t*-tests for paired data were used. Correlations were evaluated by Spearman's test. In all analyses, alpha values less than 0.05 were considered statistically significant.

## RESULTS

**Figure 1.**
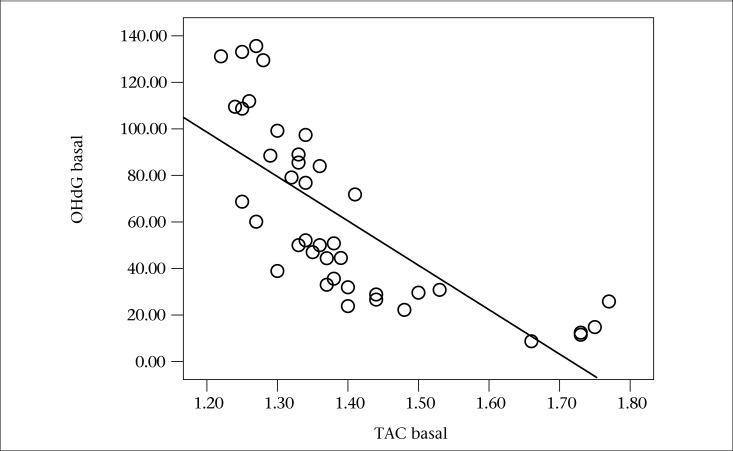
8-hydroxy-2'-deoxyguanosine (8-OHdG) and TAC levels in blood serum (r=-0.882, p<0.001) among cigarette smokers at baseline

Table 1 displays the data on age and the baseline characteristics of the two groups (smokers and never-smokers). The data from the present study indicate that the serum levels of 8-OHdG were higher (p=0.001) and that the TAC was lower (p=0.017) in smokers compared to never-smokers. As illustrated in Figure 1, there was a reverse correlation between serum levels of 8-OHdG and TAC in the smokers group (r=-0.882, p<0.001).

**Figure 2.**
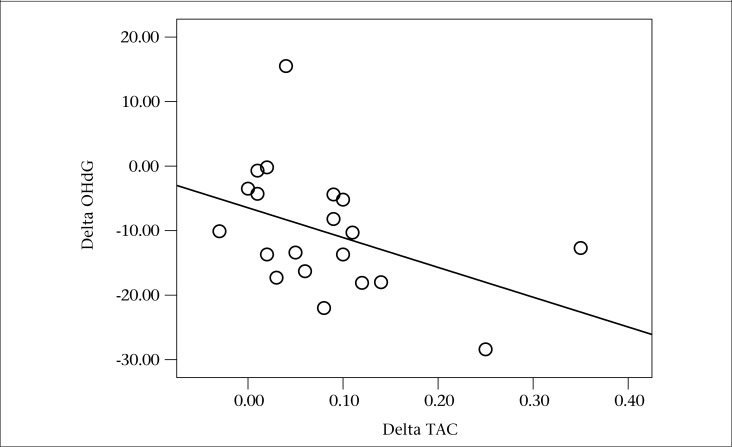
Changes in 8-hydroxy-2'-deoxyguanosine (Δ8-OHdG) and TAC (ΔTAC) serum levels between baseline and 3 months among smokers receiving FO (r=-0.540, p=0.014)

The demographic data on the cigarette smokers and the laboratory findings at baseline and for the 3 months following treatment with fish oil or placebo are presented in Table 2. There were no significant differences between the FO-treated or the placebo-treated groups in terms of mean ages or serum 8-OHdG and TAC at the beginning of the intervention. As shown, the 8-OHdG (p=0.001) level significantly decreased but serum levels of TAC markedly increased (p<0.001) after 3 months of treatment with FO. The mean decrease in 8-OHdG (i.e. mean of differences in 8-OHdG between the baseline and the 3rd month) correlated significantly with the mean increase of TAC (i.e. mean of differences in TAC between the baseline and the 3rd month in the FO-treated patients (r=-0.540, p=0.014) (Figure 2). These changes were not observed in the placebo group.

## DISCUSSION

Several studies have found lower plasma antioxidant concentrations in smokers *in vivo*. Therefore, long-term cigarette smoking is associated with oxidative stress and enhanced risks of numerous chronic diseases, including cardiovascular disease (17), cancer (21), and emphysema (3), which shorten life and impair its quality (22).

**Table 1.** Age and baseline characteristics of cigarette smokers vs never-smokers

Variable	Cigarette smokers (n=40)	Never-smokers (n=40)	p valuea
Age (years)	40.20±7.86	38.70±8.24	0.408
Serum 8-OHdGb (ng/mL)	61.82±37.77	34.92±23.83	0.001
Serum TACc (mmol/L)	1.39±0.15	1.49±0.18	0.017

^a^Cigarette smokers vs never-smokers at baseline;

^b^8-hydroxy-2’-deoxyguanosine;

^c^Total antioxidant capacity

**Table 2.** Baseline and post-treatment measures of serum 8-OHdG and TAC among cigarette smokers in fish oil intervention and placebo groups

Variable	Fish oil group (n=20)	Placebo group (n=40)	p value^a^
Age (years)	40.60±8.83	39.80±6.97	0.752
Serum 8-OHdG^b^ (ng/mL)			
Baseline	63.47±40.58	60.17±35.72	0.776
3 months	53.22±37.40	64.28±40.20
	(p=0.001)^c^	(p=0.104)^d^	
Serum TAC^e^ (mmol/L)			
Baseline	1.39±0.13	1.40±0.17	0.718
3 months	1.47±0.15	1.39±0.17	
	(p<0.001)c	(p=0.844)d

Data are mean±SD;

^a^Fish oil group vs placebo group at baseline;

^b^8-hydroxy-2’-deoxyguanosine;

^c^Before treatment vs after treatment with fish oil;

^d^Before treatment vs after treatment with placebo;

^e^Total antioxidant capacity

Previous literature has indicated, through the detection of DNA single-strand breaks, the ability of cigarette smoke to cause oxidative DNA damage (2,16). However, to our knowledge, this is the first study that examines the protective effect of fish oil supplementation on 8-OHdG serum concentration induced by cigarette smoking. Fish oil repairs oxidative damage to DNA (6). Our results support the hypothesis that cigarette smoking increases the free radical-mediated oxidative damage to DNA in smokers as shown by the significantly higher serum levels of 8-OHdG in smokers compared to never-smokers.

Several studies have found lower level of antioxidants in the blood of smokers compared to non-smokers (14,23); our data also show a decline in the circulating total antioxidant capacity of cigarette smokers.

In this study, a negative correlation between the serum levels of 8-OHdG and serum levels of TAC was seen in the cigarette smokers and the never-smokers.

The production of oxidants in quantities that overwhelm the endogenous antioxidant defense system may lead to an oxidative insult in cells and organs in cigarette smokers (14). As dietary antioxidants scavenge free radicals and other reactive species in smokers (24), it would seem that cigarette smokers would have an increased requirement for antioxidant nutrients, which may attenuate the deleterious effects of cigarette smoking (12). High intake of n-3 fatty acids diminishes the chemotactic responsiveness of neutrophils and monocytes (25) and decreases the production of free radicals (26). Some studies have concluded that there are some benefits of fish oil in oxidative damage to DNA (27-30). However, a recent study reported that EPA treatment in Atm-deficient mice led to the formation of reactive oxygen species and the accumulation of oxidative DNA damage (31).

Hong MY *et al*. reported that dietary fish oil protects against colon cancer in rats by decreasing the oxidative DNA damage, which was assessed by a quantitative immunohistochemical analysis of 8-OHdG (28). Our study is the first to reveal that high concentrations of FO reduced the 8-OHdG serum levels in cigarette smokers. On the other hand, we found in our study that FO can counteract the enhanced susceptibility of DNA to oxidative modification. Taneda S *et al*. reported that the levels of oxidative DNA damage (8-hydroxy-2’-deoxyguanosine) in the tubular cells were significantly lower in EPA-treated diabetic mice (32). EPA had a beneficial effect by suppressing the generation of reactive oxygen species. In patients with rheumatoid arthritis, Dawczynski C *et al*. demonstrated that the n-3 LC-PUFA did not increase the biomarkers of oxidative stress, such as 8-iso-PGF (2alpha) and 15-keto-dihydro PGF (2alpha) (9). DNA damage, such as that by 7, 8-dihydro-8-oxo-2’-deoxyguanosine in mildly hypertriacylglycerolemic patients (33), indicated that n-3 LC-PUFA-enriched products do not cause additional oxidative DNA damage as shown by the excretion of 7, 8-dihydro-8-oxo-2’-deoxyguanosine.

Moreover, the result of the present study supports previous studies (34) in that the administration of fish oil to the smokers ameliorates the TAC serum levels.

Likewise, our results showed a negative correlation between the changes of 8-OHdG and the TAC serum levels in the smokers treated with FO. Although this result should be confirmed, it seems that increased TAC from FO consumption could decrease 8-OHdG in smokers. Therefore, FO may exert an extra protective effect on damage to DNA in cigarette smokers.

Future investigations with larger sample-sizes that would include smokers of different ages and with different smoking histories as well as an analysis of other biomarkers of antioxidants are required to confirm this hypothesis. Accordingly, smokers could be classified by age and the nature of smoking habit (e.g. light, moderate, and heavy). Using this approach, data can be generalized to larger populations.

### Conclusions

The study provides evidence of increased oxidative stress among smokers as shown by higher serum levels of 8-OHdG and a compromised antioxidant defense system as shown by the lower serum levels of TAC. More importantly, this is evidenced by an inverse association between FO supplementation and oxidative DNA-damage in cigarette smokers.

## ACKNOWLEDGEMENTS

This work was supported by a research grant from the Biotechnology Research Center of the Tabriz University of Medical Sciences, Iran. The authors are grateful to all personnel of the Center.
